# Severe Mitral Regurgitation–Induced Acute Heart Failure due to Nonbacterial Thrombotic Endocarditis in a Patient With Urothelial Carcinoma Recurrence

**DOI:** 10.1155/crom/9938933

**Published:** 2025-01-29

**Authors:** Catarina Santos Reis, Francisco Dias, Bárbara Granja, Maria Inês Matos, Ana Ribeiro, Fernando Friões

**Affiliations:** ^1^Department of Internal Medicine, São João Local Health Unit-São João University Hospital Center, Porto, Portugal; ^2^Department of Intensive Medicine, São João Local Health Unit-São João University Hospital Center, Porto, Portugal; ^3^Department of Dermatology, São João Local Health Unit-São João University Hospital Center, Porto, Portugal

**Keywords:** acute heart failure, marantic endocarditis, nonbacterial thrombotic endocarditis, systemic embolism, urothelial carcinoma

## Abstract

Nonbacterial thrombotic endocarditis is a rare condition characterized by the formation of noninfectious vegetations on the heart valves. It is related with systemic embolic phenomena occurring predominantly in advanced cancer stages, particularly adenocarcinomas. It is a diagnosis of exclusion based on the clinical context, echocardiographic findings of a vegetation, and negative blood cultures, and distinction between infective endocarditis is challenging. We report a case of a 66-year-old woman, with history of previously treated breast and urothelial carcinoma, presenting with constitutional syndrome and pulmonary thromboembolism. Computed tomography scan revealed systemic embolic phenomena and retroperitoneal lymphadenopathies. A vegetation in the mitral valve constituted a finding on echocardiography, causing severe mitral regurgitation, which led to severe acute heart failure and culminated in patient's death. Subsequent results of immunohistochemistry of the lymph node biopsy yielded the recurrence of urothelial carcinoma.

**Summary**

- Nonbacterial thrombotic endocarditis (NBTE) is a diagnosis of exclusion that should be suspected in patients with autoimmune diseases and neoplasia in advanced stages, persistent negative blood cultures, and failure to respond to treatment in the setting of infective endocarditis (IE).
- The treatment of NBTE encompasses anticoagulation, addressing the dysfunctions encountered, and treatment of the underlying cause.
- The poor prognosis, associated with high morbidity and mortality from multiple organ dysfunction due to multiple foci of embolization, should lead the patient to be enrolled in palliative care when irreversible damage is evident.

## 1. Introduction

NBTE, also known as marantic endocarditis, is a rare condition characterized by the formation of noninfectious thrombotic lesions susceptible to systemic embolization on heart valves. Although any valve could be affected, there seems to be a predilection for the aortic and mitral valves [1]. Valvular endothelial injury is the central mechanism in the formation of platelet-rich fibrin thrombi. Specific triggers include systemic inflammatory conditions, namely, burns, sepsis or autoimmune diseases, and neoplasia, most commonly adenocarcinomas in advanced stages [1, 2].

The lack of specific signs and symptoms for NBTE makes the diagnosis deferred to postmortem autopsies. The high incidence of embolic events is the only prominent feature raising suspicion [2]. Emboli can affect any organ or territory, with cerebral and coronary circulation associated with greater morbidity [1–5]. Echocardiography has a major role in diagnosing NBTE, and the low sensitivity in detecting valvular vegetations of transthoracic echocardiography (TTE) may be overcome by using the transesophageal probe when TTE is normal or there is high suspicion of NBTE [1, 2].

Treatment recommendations are not well established. In the absence of contraindications, anticoagulation should be started immediately after a definitive diagnosis, combined with the treatment of the underlying neoplasia or systemic disease [1, 2].

Cardiac surgery is rarely indicated, being reserved to hemodynamically unstable patients, with significant valvular regurgitation and/or with the highest embolic risk [2, 3].

The prognosis depends on the underlying etiology, but it is unfavorable in patients with advanced neoplasia and with recurrent embolic phenomena, in which cases palliative treatment should be considered [1, 2].

We report a case of a patient with NBTE in the setting of recurrent urothelial carcinoma, presenting with multiple systemic embolic phenomena and severe mitral regurgitation, acute heart failure, and death. This case highlights the relationship between marantic endocarditis and urothelial carcinoma and the importance of excluding neoplastic disease in patients with similar presentation and reinforces the need of early recognition and urgent treatment institution to prevent fatal outcomes.

## 2. Case Description

A woman in her 60s was brought to the emergency department with sudden dyspnea (polypnea), fever (*T* = 38.0°C), tachycardia, a 4-month evolution constitutional syndrome of asthenia, and 10% weight loss. She was a former smoker and did not take any usual medication nor had a relevant family history. Her past medical history was relevant for left breast cancer, diagnosed 14 years ago, treated with surgery and chemoradiation with no recent evidence of recurrence, and for urothelial carcinoma diagnosed 9 years ago, treated with transurethral resection of the bladder (TURB) and intravesical immunotherapy with Bacillus Calmette–Guerin (BCG), recurring 1 year ago as squamous cell carcinoma of the ureter staging pT3NxR0 with periureteral fat invasion that required right nephroureterectomy. Follow-up with cystoscopy at 6 months showed no recurrence, and she continued surveillance visits.

**Figure 1:**
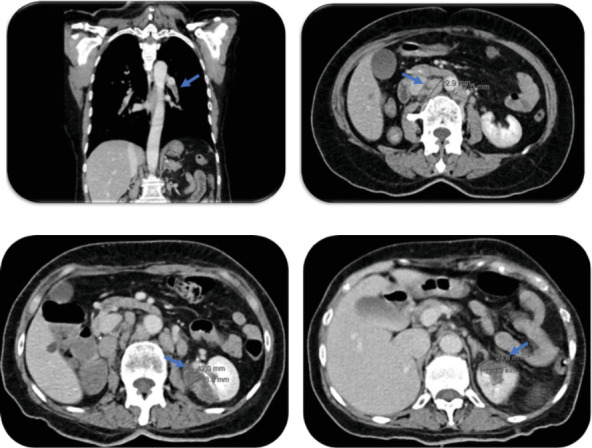
Contrast-enhanced computed tomography showing extensive bilateral pulmonary thromboembolism (a, blue arrow), retroperitoneal adenopathies, the largest located at the interaortocaval level with approximately 37 × 23 × 15 mm in diameter (b, blue arrow), areas of left kidney ischemia (c, blue arrow) and spleen (d, blue arrow).

ECG showed sinus tachycardia (110 bpm), without ST segment changes. Arterial blood gas analysis revealed hypoxemic respiratory failure. Blood tests revealed normochromic normocytic anemia (Hb 9.8 g/dL, reference interval: 12.0–16.0 g/dL), elevated D-dimers (1.71 mcg/mL, reference interval: 0–0.50 mcg/mL), B-type natriuretic peptide (BNP) of 82 pg/mL (reference interval: 100–400 pg/mL), two seriated normal high-sensitivity cardiac troponin test (5.4 ng/dL, reference interval: 0–2 ng/mL), and elevation of C-reactive protein (78 mg/dL, reference interval: < 10 mg/dL) with white blood cell count within the reference range. Thoracic CT scan showed extensive bilateral pulmonary thromboembolism extending from the right and left main pulmonary arteries to virtually all the segmental lobar branches. Color Doppler ultrasound of the lower limbs showed common femoral and right iliac deep venous thrombosis. Point-of-care TTE did not find any signs of right ventricle dilation or dysfunction. An abdominal and pelvic CT scan showed retroperitoneal lymphadenopathies, the largest at the interaortocaval level proximally to the aortic bifurcation measuring 37 × 23 × 15 mm in diameter and ischemic areas adjacent to the left kidney and spleen (Figure 1).

**Figure 2:**
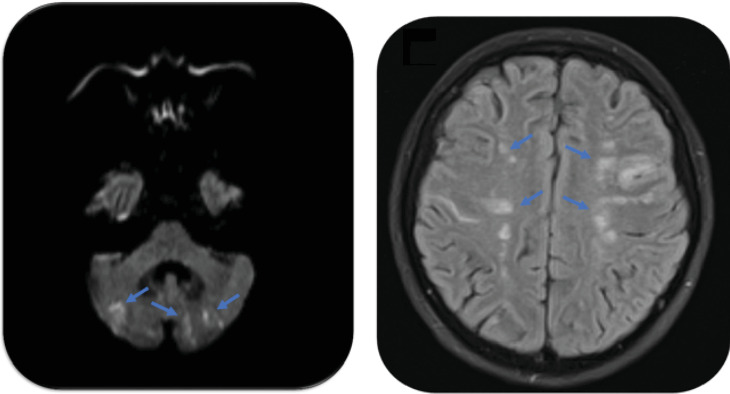
Diffusion-weighted resonance imaging showing irregular multifocal cerebral foci suggestive of small ischemic acute cardioembolic infarcts bilaterally cerebellar (a) and frontoparietal (b).

The patient responded favorably until Day 10, when she suddenly developed right hemiparesis, aphasia, and bilateral dysmetria. Although the initial CT scan was negative, diffusion-weighted cranioencephalic magnetic resonance imaging revealed multiple bilateral areas of acute ischemia of embolic origin in the cerebellum and frontoparietal lobes (Figure 2). MRI angiograms of the head and neck did not show significant stenosis in the intracranial circulation, cervical carotid, and vertebral arteries and atrial fibrillation was not detected on ECG monitoring.

Three days later, she developed sudden dyspnea and desaturation to 86% despite supplemental oxygen at 15 L/min. Lung auscultation revealed coarse crackles bilaterally. The chest radiograph showed cardiomegaly and signs of pulmonary edema. A diagnosis of acute heart failure was made, and treatment with diuretics was initiated. TTE revealed a mitral valve vegetation, and transesophageal echocardiogram (TEE) showed two irregularly shaped hypoechogenic structures measuring 8.0 × 7.0 mm each, adjacent to the mitral valve, nonmobile, leading to severe mitral regurgitation during diastole by preventing the coaptation of the cusps, and an intact interatrial septum with no evidence of shunt.

As she had not met other Duke's modified criteria for IE besides a valvular vegetation on echocardiography—she had persistently negative blood cultures and other tests (serologies and/or PCR) for other less frequent agents and absence of immunological or vascular phenomena and immunologic studies for thrombophilia yielded negative—the diagnosis of NBTE was established. Immunohistochemistry of the retroperitoneal lymph node biopsy yielded positive for urothelial carcinoma.

Anticoagulation with enoxaparin was started at admission and throughout the period of hospitalization. Considering the risk of hemorrhagic transformation from cerebral infarctions at Day 10 and deterioration of the clinical condition, heart valve surgery was not an option, and the patient continued anticoagulation and started antibiotic therapy. Antibiotic was discontinued as the patient had persistently negative blood cultures and no evidence of infection. Finally, after concluding that clinical deterioration and complications were irreversible, palliative care was offered to the patient.

The patient died 1 week after experiencing multiple systemic embolic events and severe mitral regurgitation, which lead to severe acute heart failure.

## 3. Discussion

The causal association between neoplasia and thrombi formation has long been known, since 1865, when Trousseau described migratory thrombosis as the first manifestation of an occult gastric neoplasia. The interaction between macrophages, monocytes, and neoplastic cells releases interleukins, tumor necrosis factor (TNF), and tissue factors that cause endothelial damage. Macrophages also overactivate the coagulation cascade. Endothelial damage leads to the onset of platelet aggregation culminating in thrombogenesis and hypercoagulability. Around half of cancer patients have embolic events, mainly in the coronary, splenic, renal, cerebral, and mesenteric arterial circulation [3].

Systemic embolism is a devastating and potentially fatal complication of infective endocarditis that occurs in up to 50% of cases, with the central nervous system being the most common, followed by spleen, kidney, lung, liver, bone and joint, iliac artery, and mesenteric artery [4]. NBTE is a rare manifestation of arterial thromboembolism whose incidence is unknown, being more frequently reported as a postmortem finding occurring in approximately 1.6% of autopsies. Around 80% of cases occur in neoplasms in advanced stages, mostly adenocarcinomas of unknown origin, followed by lung, gastric, and pancreatic carcinomas [1–3].

In our case report, the associated cancer with NBTE is an infrequent association [2]. Another singularity of this case is the development of multiple systemic embolic events in a short period of time, in which cancer recurrence was unknown, presenting initially as pulmonary thromboembolism and peripheral venous thrombosis with asymptomatic splenic and renal infarction. Embolic phenomena affecting the pulmonary and venous circulation are less frequent in patients with NBTE, as seen in our case, and later cerebral manifestations, occurring in about 40% of cases, are more frequently seen in NBTE (33%) comparative to IE (19%) [1–3].

The last event observed in our report was severe mitral regurgitation culminating in acute heart failure. NBTE rarely presents with symptoms of valvular dysfunction or acute heart failure, and the cases reported in the literature are associated with the aortic and not mitral valve. Furthermore, factors such as an advanced age, presence of antiphospholipid syndrome, splenic or renal infarction, pulmonary or peripheral thromboembolism, myocardial infarction, and mitral valve regurgitation are all associated with a higher risk of in-hospital mortality, and the vast majority were present in our patient and inevitably contributed to her death [5].

TTE is undoubtfully useful for the diagnosis, but sensitivity can be affected considering that vegetations are friable and decrease in size or disappear after embolization, due to the scarce inflammatory reaction at the insertion site, yielding false-negative results [1, 2]. TEE should be the imaging method of choice when TTE is normal or when there is a strong suspicion of NBTE, considering its greater diagnostic sensitivity [1, 2]. The major role of TEE was emphasized in our clinical case given the normality of the initial TTE performed.

As an isolated finding, a vegetation on echocardiography does not permit the distinction between infectious or noninfectious etiology, and the diagnosis is exclusively confirmed by histochemical analysis. Nevertheless, in NBTE, vegetations are generally smaller (normally < 10 mm); have irregular edges, wider base, and heterogeneous echodensity; and are easily friable. They adhere to the valvular coaptation surface, without valvular destruction, and the motion they present is not independent on the cardiac valve motion [2]. Echocardiographic findings compatible with those described were present in our case.

The literature also emphasizes the role of modern neuroimaging techniques, such as diffusion-weighted magnetic resonance imaging, as a useful tool in differentiating stroke patterns between NBTE and IE. As observed with our case, patients with NBTE have areas of cerebral infarction in multiple territories, widely distributed, and of various sizes, considering the greater potential for fragmentation of the vegetations in these cases [1, 3].

Treatment recommendations include the initiation of anticoagulation soon after the recognition of this entity and for life in the absence of contraindications, combined with the treatment of the underlying cause. Regarding the best therapeutic approach, this involves the use of heparin, given the decrease in the occurrence of thromboembolic complications, especially in patients with cancer. Vitamin K antagonists proved to be less effective in reducing recurrent embolization, and direct oral anticoagulants are potential alternatives, but there is still a lack of scientific evidence to recommend their use [2]. As evidenced by our case, recurrence of embolism can occur despite treatment. Surgical treatment has a limited role in the management of NBTE, and there are no specific guidelines for these patients. However, it should be considered in patients who experience thromboembolic events despite adequate anticoagulation treatment and signs and symptoms of acute heart failure or acute valve rupture. The risks and benefits of the intervention must be balanced against the prognosis and life expectancy of the underlying disease. The prognosis is often poor, considering the advanced cancer stages and the recurrence of systemic embolisms, which in conjunction carry high morbidity and mortality rates [2, 3].

## 4. Conclusion

In summary, we described a case of recurrent embolism due to NBTE in a patient with recurrence of an infrequently reported malignancy. When the diagnosis of IE is suspected but blood cultures and other microbiologic tests are negative, when there is no response to antibiotic treatment and a hypercoagulable state is identified, the diagnosis of NBTE should be strongly considered. The use of TEE should be mandatory for detection of vegetation, considering the low sensitivity with TTE. Although very rare, valvular dysfunction can occur and be life-threatening in NBTE.

The cornerstone of the management remains anticoagulation and treatment of the underlying cause. In our case, considering multiple organ dysfunction due to multiple foci of embolization and the associated morbidity, directed therapy focusing on cancer or cardiac dysfunction was not possible, and the patient was enrolled in palliative care.

The scarcity of data about the prevalence of NBTE and the best diagnostic and therapeutic strategies as well as poor clinical outcomes is an urgent call for future investigations.

## Data Availability

The data that support the findings of this study are available from the corresponding author upon reasonable request.
